# Optimizing Antimicrobial Drug Dosing in Critically Ill Patients

**DOI:** 10.3390/microorganisms9071401

**Published:** 2021-06-28

**Authors:** Pedro Póvoa, Patrícia Moniz, João Gonçalves Pereira, Luís Coelho

**Affiliations:** 1 Polyvalent Intensive Care Unit, Sao Francisco Xavier Hospital, CHLO, 1449-005 Lisbon, Portugal; patricia.moniz25@gmail.com (P.M.); luismiguelcoelho16@gmail.com (L.C.); 2 Comprehensive Health Research Centre (CHRC), Nova Medical School, New University of Lisbon, 1169-056 Lisbon, Portugal; joaogpster@gmail.com; 3 Center for Clinical Epidemiology and Research Unit of Clinical Epidemiology, OUH Odense University Hospital, 5000 Odense, Denmark; 4 Intensive Care Unit, Vila Franca de Xira Hospital, 2600-009 Vila Franca de Xira, Portugal

**Keywords:** pharmacokinetics, antibiotics, sepsis

## Abstract

A fundamental step in the successful management of sepsis and septic shock is early empiric antimicrobial therapy. However, for this to be effective, several decisions must be addressed simultaneously: (1) antimicrobial choices should be adequate, covering the most probable pathogens; (2) they should be administered in the appropriate dose, (3) by the correct route, and (4) using the correct mode of administration to achieve successful concentration at the infection site. In critically ill patients, antimicrobial dosing is a common challenge and a frequent source of errors, since these patients present deranged pharmacokinetics, namely increased volume of distribution and altered drug clearance, which either increased or decreased. Moreover, the clinical condition of these patients changes markedly over time, either improving or deteriorating. The consequent impact on drug pharmacokinetics further complicates the selection of correct drug schedules and dosing during the course of therapy. In recent years, the knowledge of pharmacokinetics and pharmacodynamics, drug dosing, therapeutic drug monitoring, and antimicrobial resistance in the critically ill patients has greatly improved, fostering strategies to optimize therapeutic efficacy and to reduce toxicity and adverse events. Nonetheless, delivering adequate and appropriate antimicrobial therapy is still a challenge, since pathogen resistance continues to rise, and new therapeutic agents remain scarce. We aim to review the available literature to assess the challenges, impact, and tools to optimize individualization of antimicrobial dosing to maximize exposure and effectiveness in critically ill patients.

## 1. Introduction

One of the recommendations from the Surviving Sepsis Campaign (SSC) is antibiotic therapy in the first hour [1]. This is a key element for successful sepsis management. However, for this to be effective, several decisions must be addressed simultaneously when prescribing antimicrobials (AM): (1) AM choices should be adequate, covering the most probable pathogens; (2) they should be administered in the appropriate dose, (3) by the correct route, and (4) using the correct mode of administration to achieve successful concentration at the infection site.

It is well known that inadequate empirical antibiotic therapy is associated with poor outcomes. However, there are scarce data concerning the impact of inadequate dosing on outcomes of critically ill patients [2,3]. Moreover, patients with sepsis and septic shock present an increased risk of underdosing, increased volume of distribution (Vd), increased clearance, risk of overdosing, and risk of renal and hepatic failure. In addition, we are facing infections frequently caused by pathogens with higher minimum inhibitory concentrations, consequently increasing the risk of inadequate dosing. In order to be effective, AM dosing should be optimized to quickly attain bactericidal concentrations at the infection site. To optimize the AM exposure of pathogens, it is also fundamental to consider drug penetration in different organs both in health and disease [4,5].

In addition to the impact on clinical outcomes, optimizing AM dosing could decrease the emergence of resistance and in doing so prolong the lifespan of currently available AM. This review covers pertinent topics for AM dosing optimization, including relevant AM pharmacokinetics and pharmacodynamics, specific dosing strategies, and respective outcomes, focusing on the most frequently prescribed AM in the intensive care setting.

## 2. Pharmacokinetic and Pharmacodynamic Characteristics of Antimicrobials

The therapeutic window of a drug is defined according to previously studied dose–response relationships which will also determine the limits of safe concentration and dosage. The dose and duration of dosing intervals of AM are determined according to their pharmacokinetic/pharmacodynamic (PK/PD) properties [6]. However, in critical illness, multiple underlying derangements provoke pathophysiological alterations that change the PK/PD of drugs and therefore provoke dynamic changes in drug concentration, as will be further mentioned in detail. 

Pharmacokinetic parameters define the concentration–time course of the AM and are based on the principles of absorption, distribution, metabolism, and elimination, all of which are greatly affected by critical illness [6,7]. Absorption reflects bioavailability and depends on both drug characteristics, such as physicochemical properties, and tissue/organ characteristics. Absorption issues in the critically ill usually promote intravenous drug administration, guaranteeing 100% bioavailability [6,8,9]. A detailed analysis of PK alterations in the critically ill will be further discussed.

The most important PK parameters include area under the plasma concentration time–curve (AUC 0–24 h), peak plasma concentration (Peak), and the trough concentration prior to the upcoming dose (Cmin) [6]. These PK parameters, along with respective dosage strategies and physicochemical characteristics, will determine serum drug concentrations and consequently the concentration in body fluids and tissues [10].

Pharmacodynamics reflect parameters of AM activity [11]. In other words, PD determines the relationship between AM concentration and the respective effect on target pathogens, relying on the minimal inhibitory concentration (MIC), which reflects the in vitro susceptibility of the pathogen [6]. Therefore, PD connects the PK exposure (serum concentration) to the drug’s pharmacological (killing or growth inhibition capacity) and toxic effects [10]. In theory, when the MIC increases, PK exposure should do the same to guarantee an optimal PK/PD index [11]. 

Antimicrobials are classified according to their dose–response relationships into the following PK/PD groups: time-dependent, concentration-dependent, and concentration-dependent with time-dependence [6,12]. The effect of time-dependent AM, such as β-lactams, depends on the cumulative percentage of time over 24 h by which the free AM concentration exceeds the MIC (%fT > MIC). The killing rate does not improve if concentration greatly exceeds the MIC [13]. In concentration-dependent AM, such as aminoglycosides (AG), their effect depends on the peak concentration divided by the MIC (Peak/MIC). The higher the AM concentration, the greater the extent and rate of bactericidal activity [10]. Optimal Peak/MIC targets for AG will be further discussed in this review. The effect of concentration-dependent drugs with time-dependence, such as fluoroquinolones and glycopeptides, is determined by the AUC 0–24 h divided by the MIC, and specific targets, as will be further discussed, depend on the AM [6,10]. 

**Figure 1:**
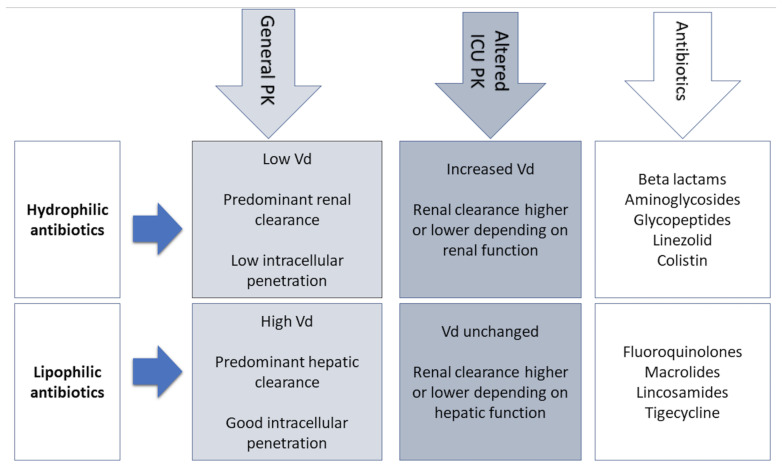
Physicochemical properties of antibiotics. Vd—Volume of distribution; PK—Pharmacokinetics.

The physicochemical properties of AM should also integrate the choice of appropriate dosing (Figure 1). According to their physicochemical properties, AM are categorized as hydrophilic (aminoglycosides, β-lactams, and glycopeptides) and lipophilic (fluoroquinolones, macrolides, lincosamides) [12,14]. Hydrophilic AM are characterized by tissue distribution limited to the extracellular space with the majority depending on renal clearance. However, lipophilic AM have an intracellular accumulation and depend on hepatic clearance. Consequently, hydrophilic, more so than lipophilic drugs, are significantly affected by the PK alterations of critical illness, especially in cases of sepsis in which renal function fluctuations and Vd expansion, due to capillary leakage and volume resuscitation, are common [6,15]. This will determine the need for adjustments of loading and maintenance doses of hydrophilic AM in the critically ill patients [6]. Furthermore, the serum concentration of AM depends on their dosing, bioavailability, and Vd. Hydrophilic AM, having an extracellular distribution, have low Vd, while lipophilic AM have high Vd due to their rapid cellular uptake [16,17].

In the ICU patient, traditional dosing strategies will most likely be insufficient to achieve the desired PK/PD targets of maximal AM activity. Therefore, an individualized approach considering specific MICs and regimens most likely to attain PK/PD goals can provide reasonable solutions.

## 3. PK Changes in Critically Ill Patients

Critically ill patients present important pathophysiological changes that significantly modify the PK of antimicrobials [7]. In septic shock, blood flow of gastrointestinal tract and subcutaneous tissue are severely reduced and shunted to vital organs such as the brain and heart, compromising reliable drug absorption with administration via these routes. As a result, intravenous administration of AM is always recommended in patients with sepsis and septic shock [1,7].

Patients with sepsis and septic shock present a significant fluid shift from the intravascular compartment to interstitial space due to endothelial damage and increased capillary leak. This leak results in severe hypotension requiring aggressive intravenous volume resuscitation that further increases the Vd, the eventual simultaneous prescription of vasopressors, and development of organ failures such as circulatory shock and renal failure (Table 1). For these reasons, hydrophilic AM (aminoglycosides, β-lactams, glycopeptides, and lipopeptides) with an extracellular distribution need a higher loading dose to achieve therapeutic concentrations [3,18]. On the other hand, the Vd of lipophilic antibiotics is not significantly influenced by these changes and does not require dose adjustments [19]. 

Another important factor that can influence the Vd of AM is the modification in protein binding. Since albumin is the main plasma-binding protein for many AM (e.g., cefazolin, ceftriaxone, ertapenem, and daptomycin), its decreased concentration in septic patients has a direct impact on the PK of antibiotics [20]. With low plasma albumin, there is an increase of the unbound antibiotic, increasing its Vd and clearance, and leading to lower and probably suboptimal AM concentrations toward the end of dosing intervals. For these reasons, therapeutic drug monitoring (TDM) should include an adjustment for low albumin levels or a direct measurement of free drug levels [21].

Acute kidney injury (AKI) is a common complication of septic shock that leads to a reduction of glomerular filtration rate (GFR) and consequently decreased drug clearance. However, the evaluation of renal clearance in these patients is quite difficult due to frequent changes in fluid balance and fluctuations of renal function [22]. The use of serum creatinine levels to estimate GFR is very inaccurate and could lead to both under- and overestimations. However, no other method, including 24 h urine collections, has proved to be better for GFR estimation [23]. Consequently, alternative methods for the assessment of renal function are warranted, such as the measurement of renal biomarkers (e.g., cystatin C), measuring creatinine clearance (CrCl) with urine samples collected in short intervals (4–6 h), and kinetic GFR equations that take into account the magnitude of serum creatinine changes relative to baseline [24,25,26].

A significant number of septic patients with AKI require renal replacement therapy (RRT). Several modalities of RRT are available, including intermittent hemodialysis, ultrafiltration, hemofiltration, and hemodiafiltration. Drug clearance is affected by the properties of the membrane used and other factors such as pore size, sieving coefficient, the surface area of the filter membrane, the dialysate flow rate, or the duration of dialysis [27]. Hydrophilic drugs are easily cleared by dialysis (small molecules, such as β-lactam and aminoglycoside), whereas large molecules (>1000 Da), such as vancomycin, are poorly cleared [28].

Equally important is the presence of augmented renal clearance (ARC), especially for antibiotics eliminated by the kidney, such as β-lactams, carbapenems, and glycopeptides [29]. Augmented renal clearance causes a significant decrease in serum antibiotic levels to non-therapeutic levels that can compromise the efficacy of antibiotic therapy. As a result, it is frequently necessary to markedly increase the doses of AM to achieve therapeutic targets [30].

## 4. Antibiotics in the ICU

### 4.1. Β-Lactams 

The β-lactams’ broad spectrum of AM activity and low toxicity profiles unsurprisingly render them first-line options in serious infections, namely Gram-negative bacilli (GNB) infections, and the most commonly prescribed AM in critical care [14,31]. The authors have focused on the review of relevant PK/PD aspects and dosing optimization of the three main β-lactams in critical care: penicillins, cephalosporins, and carbapenems.

Β-lactams are generally hydrophilic, with low Vd, moderate to low protein binding, and essentially renal excretion [15]. In vivo animal studies have clearly shown that β-lactams are characterized by a slow continuous kill, in other words, time-dependent bactericidal activity [32,33]. Consequently %fT > MIC is the optimal PK/PD parameter for β-lactams, with the recommended interval of 40–70% [15,34], varying according to the AM and underlying pathogens [35]. This time-dependent effect is independent of peak values and little post-antibiotic effect exists, except for carbapenems [36]. Since β-lactams have short or no post-antibiotic effect, when AM concentration falls below the MIC at the infection site, residual pathogens can rapidly regrow [16]. Furthermore, frequent Vd and Cl alterations accentuate the risk of suboptimal drug concentrations in the face of critical illness [15]. For example, with hypoalbuminemia, highly protein-bound β-lactams such as ceftriaxone, ertapenem, flucloxacillin, and oxacillin will present increased free fractions [37]. 

Recommended %fT > MIC varies according to the different β-lactams, consequently reflecting differences in killing rates. In GNB infections, %fT > MIC >50% for piperacillin-tazobactam, >70% for ceftazidime and cefepime, and >40% for carbapenems are considered optimal targets, confirming the fastest killing rates with carbapenems and the slowest with cephalosporins [10]. Nevertheless, various studies point to insufficient β-lactam dosing strategies in the critically ill septic patients [36,38,39]. 

Initial loading doses followed by prolonged infusions have been associated with higher PK/PD target attainment rates and better clinical outcomes [15,40]. Several studies in the critically ill tend to promote the use of extended infusions (prolonged such as 40–50% of the dosing interval or continuous) for the attainment of desired PK/PD targets, instead of standard bolus dosing [41,42,43,44,45,46]. In a recent meta-analysis, Vardakas et al. compared the effect of prolonged with short-term infusions of anti-pseudomonal β-lactams on the mortality of septic patients. Reduction of mortality was observed with prolonged infusions compared with short-term infusions, although clinical cure was not higher. The small number of randomized clinical trials (RCT) that analyzed both efficacy and mortality might justify this finding. Moreover, the definition of clinical cure itself might be compromised, considering that data of microbiological eradication were absent in many studies. Dosing issues, such as suboptimal dose administration in nosocomial pneumonia, can also underestimate the effect of prolonged infusions [40]. Nevertheless, much uncertainty remains regarding the ideal dosing strategy of β-lactams, without clear outcome benefits linked to continuous infusion administration [47]. 

Vardakas et al. in a recent metanalysis found that prolonged infusions of cephalosporins were not associated with additional benefit or reduced mortality when compared with short-term infusions [40,48]. Current dosage recommendations have also been found to be insufficient, such as with ceftazidime regimens, especially in patients with higher GFR requiring increased dosing regimens. Georges et al. found that with a ceftazidime loading dose of 2 g followed by continuous infusion, a steady state was achieved faster than that with discontinuous administrations. Moreover, in polytrauma patients, achieving a steady state took longer, which is a finding most likely attributable to the higher Vd of these patients [38]. 

The consumption of carbapenems has been increasing worldwide in response to an increasing prevalence of multidrug-resistant (MDR) GNB. However, GNB that classically maintained susceptibility to carbapenems in the ICU, such as *Acinetobacter baumanni*, have shown worrisome rates of resistance to carbapenems and have been associated with carbapenem misuse [49]. As previously stated, the optimal PK/PD target for carbapenems is %fT > MIC >40% [10]. However, even in patients with normal renal function, attainment of this target is suboptimal and decreases to 65% with a MIC of 4 ug/mL [50]. While prolonged infusions for cephalosporins were not found to be beneficial from an outcome perspective, Vardakas et al. associated increased survival with prolonged infusions when β-lactam/β-lactam inhibitor combinations and carbapenems were analyzed separately [40].

More aggressive dosing regimens of β-lactams in attempts to overcome frequent PK changes risk increases in toxicity, stimulating the implementation of TDM of this class of AM [15,51,52]. Consequently, TDM for β-lactams is currently recommended as routine implementation in the treatment of critical illness, although it is not well standardized [15]. Steady-state Cmin sampling is currently advocated. However, dosing software could contribute to further optimization [53].

Regarding toxicity, β-lactams are usually well tolerated, with a high safety profile. Although myelosuppression and neurotoxicity are some of the more common adverse effects, especially in higher doses, specific toxicity thresholds have not been determined [15,54]. However, neurotoxicity is the most commonly reported adverse effect and occurs most frequently with the following β-lactams: benzylpenicillin, imipenem, cefepime, and ceftazidime [55,56,57].

With the disconcerting surge of MDR GNB, new β-lactams have been recently approved for nosocomial pneumonia as well as complicated urinary tract and abdominal infections. Ceftolozane, a novel cephalosporin, can be combined with the well-known β-lactamase inhibitor, tazobactam, with good anti-Pseudomonas activity. Ceftazidime-avibactam, a combination of a known cephalosporin with a non-β-lactam β-lactamase inhibitor, is a new agent against *Klebsiella pneumoniae* carbapenemase carbapenem-resistant Enterobacteriaceae. However, studies in the critically ill are very scarce [58,59,60]. 

### 4.2. Aminoglycosides

Aminoglycosides are frequently prescribed as empirical therapy regimens in septic ICU patients, namely when suspicion of GNB infection prevails [61]. Furthermore, recent guidelines recommend combination therapy in septic shock [1]. The rationale for combination therapy originated from in vitro findings of synergistic bactericidal activity with certain combination therapies in the context of *Pseudomonas aeruginosa* and other GNB infections [62,63,64]. However, in a recent metanalysis comparing β-lactam monotherapy with β-lactam/aminoglycoside combination therapy, evidence regarding non-neutropenic septic patients does not show a mortality benefit with combination therapy [65]. 

Aminoglycosides are hydrophilic, with low Vd and drug clearance proportional to GFR [15]. Extended-interval dosing of a high, single dose is recommended in GNB infections. 

The bactericidal activity of AG is observed with concentration-dependent patterns [66]. Therefore, AG efficiency is reflected in the PK/PD patterns of Peak/MIC and AUC/MIC [10]. Ideally, the peak concentration to MIC ratio (Peak/MIC) should reach the target of eight to 10 times the MIC [67]. AG demonstrate a considerable post-antibiotic effect (PAE), which is defined as the time needed for microbial regrowth following AM removal. Consequently, after short exposure to AG, bacterial growth suppression persists, with greater AG concentration leading to greater PAE [68,69]. Therefore, sub-MIC trough levels can be tolerated and less-fractioned dosing regimens can be favored for these concentration-dependent AM [16].

Aminoglycosides TDM is recommended for routine evaluation in the critically ill [15] and is encouraged to optimize re-dosing and reduce toxicity, especially with trough concentration monitoring [61]. However, standard dosing regimens have been associated with lower peak concentrations in up to 40% of ICU patients [70]. For example, higher dosing regimens for amikacin and gentamicin in critically ill patients have recently been recommended to surpass suboptimal dosing [71]. 

Although routinely recommended, TDM practices vary among countries, as demonstrated in the recent cohort AMINO III study which evaluated different AG dosing and monitoring strategies in ICU patients. ICU AG regimens for sepsis were mainly comprised of amikacin (two-thirds), followed by gentamicin (one-third) and rarely tobramycin. However, aminoglycoside preference may vary amongst countries, and the high prevalence of French ICUs in this study may justify the frequent prescription of amikacin. Most patients received AG as combination therapy, namely in association with β-lactams. Median AG therapy duration was 2 days with two doses per course and 26 h dosing intervals. More than half received a second dose and achieved a PK/PD target of Peak/MIC > 8. Although gentamicin was less frequently prescribed, target attainment was also lower than for amikacin (59% versus 71%, respectively) [61]. An increased Vd of gentamicin has been reported in sepsis, consequently leading to suboptimal gentamicin exposure and treatment failure [72]. These findings justify higher gentamicin loading doses in the critically ill [73]. Although the “off-label” strategy of extended interval of high doses of AG was frequent, one-fifth of patients persisted with acute kidney injury at the end of AG treatment [61]. 

The frequency of AKI attributable to AG therapy is difficult to determine, especially in septic shock patients in whom AKI is frequent [74]. The AMINO III study failed to correlate peak target achievement with clinical outcome, possibly reflecting the impact of effective β-lactam combination therapy and consequently minimizing the impact of AG target attainment failure [61]. 

### 4.3. Glycopeptides

#### Vancomycin

Most published data regarding vancomycin dosing and TDM are retrospective observational or PK/PD assessments, with few published RCT. Since the 2009 guidelines on the treatment of serious methicillin-resistant Staphylococcus aureus (MRSA) infections, new light has been shed regarding the efficacy and safety of previous recommendations [75]. Issues such as dosing strategies in obese patients, safety profiles in daily dosages exceeding 3 g, continuous infusion strategies, and renal failure are some examples where insufficient data precluded adequate coverage. Moreover, existing recommendations of exposure effectiveness are based mainly on studies of MRSA bacteriemia, with fewer studies of pneumonia and endocarditis. Nevertheless, much controversy around vancomycin dosing and TDM still exists [75].

Vancomycin is hydrophilic, has a low Vd, and elimination is mainly renal. Altered Vd and drug clearance, namely ARC, in the critically ill may lead to low drug exposure [15]. 

The AUC 0–24 h/MIC ratio > 400 is considered the optimal PK/PD “efficacy” index due to findings of bactericidal activity close to or exceeding this value. Lower values seem to promote MRSA resistance and vancomycin-intermediate strains [76,77]. However, the current formulas of estimating GFR risk imprecise estimations for AUC determinations [78], especially in the presence of significant interindividual variability of exposure profiles [79,80]. Nevertheless, in cases of serious invasive MRSA infection, for maximum efficacy and minimal nephrotoxicity, current guidelines recommend daily AUC 400–600 mg/h/L, assuming an MIC of 1 mg/L [75]. In cases of septic shock, higher exposures are advocated [81,82,83]. Upon culture results excluding invasive MRSA infection, de-escalation is recommended, with either the reduction or elimination/substitution of vancomycin [75].

In the critically ill with normal renal function, a loading dose of 25–30 mg/kg is recommended. Studies comparing intermittent dosing versus continuous infusion demonstrated equivalence between both strategies [84]. With intermittent dosing strategies, subsequent administrations should consist of 15–20 mg/kg every 8–12 h. Continuous infusions are also acceptable dosing strategies that have been linked to lower toxicity [85] and have gained preference due to practical reasons and facilitation of TDM [15]. 

TDM is recommended in the critically ill and traditionally has been based on the monitoring of trough concentrations, namely due to a more practical clinical approach when compared to AUC measurements. The 2009 IDSA guidelines recommend Cmin of 15–20 mg/L as a surrogate for AUC/MIC [86]. However, the clinical benefits of higher trough values have not been convincingly documented. Recent studies suggest that trough concentration alone may not be an adequate substitute for AUC surveillance and may underestimate actual vancomycin exposure [78,87,88,89]. 

Patel et al. characterized the exposure–response profiles of the dosing regimens proposed in the 2009 IDSA guidelines. Although target attainment was improved for MIC of 1 mg/L when compared to higher MIC values, it presented high variability when daily doses of up to 2 g were used. At least 3 g daily allowed an 80% target attainment, albeit at the expense of higher toxicity, especially amongst critically ill patients. As for the recommended use of trough values for effect surveillance, Cmin between 15 and 20 mg/L were not always necessary for AUC/MIC > 400 with an MIC of 1 mg/L and was not consistent with adequate AUC/MIC with higher MIC. The discordance between trough and AUC values is easily explained by the fact that trough values represent drug exposure at the end of the dosing interval, whereas the AUC represents the average 24 h drug concentration [90]. Consequently, isolated trough values can prove insufficient for adequate vancomycin dosing guidance. 

Regarding vancomycin´s toxicity profile, AKI constitutes the main dosing concern. Most cases of vancomycin-associated AKI occur after the 4th day of therapy, and increased risk has been associated with both trough concentrations (greater than 15–20 mg/L) [91] and along the AUC continuum [75]. Neely et al. in a prospective trial found that AKI was associated with median trough concentrations of 15.7 mg/L and AUC of 625 mg/h/L [92]. 

Although current evidence supports the aforementioned PK/PD targets with the respective efficacy and toxicity profiles, the emergence of more reliable data of multicenter, randomized, and dose-optimized outcome trials may change the way we approach vancomycin dosing [75]. 

### 4.4. Colistin

Current guidelines for ventilator-associated pneumonia (VAP) recommend empirical combination therapy with colistin and another antipseudomonal AM in ICUs where carbapenem-resistant (CR) GNB are highly prevalent [93,94]. Recent meta-analyses evaluated the efficacy and safety of colistin for VAP caused by MDR GNB and found it to have similar efficacy and safety as seen with β-lactams. However, multiple limitations of the studies included call into question the strength of these findings [95,96,97]. 

A recent RCT conducted by Cisneros et al. [98], the Magic Bullet study, sought to compare the efficacy and safety of empirical colistin versus meropenem in late VAP occurring in ICUs with a high prevalence of CR-GNB. The findings did not favor empirical use of colistin in late VAP. Due to high rates of nephrotoxicity in the colistin group, the trial was stopped, and half of the desired sample size was analyzed. Both groups presented similar mortality rates (22.5% in the colistin group; 21.4% in the meropenem group) and poor microbiological cure (56%). Colistin was associated with significantly higher nephrotoxicity and noninferiority was not shown because of early termination of the trial [98]. 

Colistin is administered as the prodrug colistin methanesulfonate (CMS), which has a low Vd, mixed elimination, protein binding of 59–74%, and concentration-dependent bactericidal activity. The recommended PK/PD index for efficacy is the free AUC 0–24/MIC (fAUC0–24/MIC), with ratios of 10.9–13.7 for optimal killing of *Pseudomonas aeruginosa* and 3.5–9.0 for the killing of *Acinetobacter baumannii* [99,100]. 

A loading dose should be administered to compensate large Vd and dosing regimen adapted to renal function. Dosing can be challenging when variability exists in the conversion of the prodrug to the active compound, especially in increased Cl. Recent studies recommend a single dose for all Cl values > 90 mL/min [101].

Routine TDM is not currently strongly recommended nor discouraged. In the context of ongoing conversion of the prodrug, the therapeutic index is narrow, and trough concentration should be attained when conversion and consequently CMS concentrations are lowest (immediately before the next drug administration). A steady-state concentration of 2 mg/L is associated with efficacy and limited nephrotoxicity [101,102]. This should correspond to an fAUC0–24/MIC of 12 (for MIC < 2 mg/L). Further research is needed to correlate clinical outcomes [15]. Neuro- and nephrotoxicity are associated with prolonged high exposure. Trough concentration >2.4 mg/L increases the probability of nephrotoxicity [15,103,104,105,106]. 

### 4.5. Fluoroquinolones

The AM spectrum of fluoroquinolones includes GNB, Gram-positive, and with some, also anaerobic coverage, with popular use since the 1980s [107]. However, over the years, GNB resistance has drastically increased, with susceptibility rates less than 70% for agents such as *Escherichia coli*, *Pseudomonas aeruginosa*, and *Proteus mirabilis* [107,108]. Moreover, the frequent and inappropriate use of this class has been associated with *Clostridium difficile* infection outbreaks and the emergence of MRSA [109,110]. The issues related to fluoroquinolone resistance have led to their infrequent use as first-line AM in the ICU setting where GNB such as *Pseudomonas aeruginosa*, *Acinetobacter baumannii*, and *Stenotrophomonas maltophilia* are often fluoroquinolone-resistant. However, when used in the ICU setting, fluoroquinolones should be administered at maximum doses (levofloxacin 750 mg every 24 h; ciprofloxacin 400 mg every 8 h) [107]. 

Fluoroquinolones are lipophilic, have moderate to low protein binding, elimination is renal only in some degree, and Vd is usually unaffected in the critically ill. Bactericidal activity against GN is concentration-dependent, whereas activity against GP such as *Streptococcus pneumoniae* is less so, classifying their killing activity as both concentration and time-dependent. Optimized dosing should be evaluated to increase AUC0–24 h/MIC and/or Peak/MIC ratios. Overall, the AUC0–24 h/MIC seems to be the best predictor of fluoroquinolone efficacy [15,111,112].

Forrest et al. in 1993 characterized microbiological and clinical cure according to ciprofloxacin AUC/MIC in serious GNB infections, concluding that AUC/MIC targets ≥125 were associated with better outcomes [113]. Zelenitsky et al. in a recent study found that higher AUC/MIC targets (>250) were associated with improved clinical outcomes in serious Enterobacteriaceae infections when compared to the conventional AUC/MIC cut-off >125. However, the susceptibility MIC profiles was associated with higher AUC/MIC ratios albeit with a limited distribution of lower values, possibly justifying the lack of statistical significance at the conventional breakpoint of 125 [114]. 

Currently accepted PK/PD targets depend on the underlying pathogen. For Gram-positive infections, a AUC0–24/MIC 25–30 target is recommended, contrary to the higher AUC/MIC >125 for GNB [113,114]. Pertinent adverse effects in the ICU setting include neurotoxicity (seizures, altered mental status), associated *C. difficile* infection, prolongation of QTc interval, and altered serum glucose control [107,115,116,117]. However, toxicity thresholds have not been specified [15].

TDM is debatable but may be useful (due to inter-individual PK variability in ICU and emergence of resistance), particularly where MIC is close to susceptibility breakpoint [15].

## 5. Strategies to Optimize Dosing

There is increasing evidence that front-line antibiotic inappropriateness is common and may have significant impact on the outcome of patients with severe infections and septic shock [31,36]. Large spectrum AM as well as combination therapy have both been proposed as strategies to enlarge antibacterial spectrum and improve patient outcomes [1]. However, appropriate spectrum of antibiotic therapy may be insufficient if adequate exposure is missed [3,118]. Early achievement of adequate antibiotic concentration is of paramount importance when treating patients with septic shock.

However, the concept of adequate dosing is ill-defined. Antibiotics are usually prescribed in a traditional pattern, using the conventional dose, both in the empirical setting and as directed therapy [2], taking only into account renal and liver function along with microorganism susceptibility pattern. Moreover, dose is usually maintained throughout treatment course, despite the PK changes.

As already pointed out, several changes occur in infected critically ill patients that often lead to significantly different antibiotic exposures, impacting effectiveness and toxicity. Moreover, bacteria inoculum, commonly much higher in critically ill patients [119], may also influence the bacteria killing kinetics [120] and may contribute to therapeutic failure. Consequently, dose individualization, according to the PK of AM and the patient’s unique clinical characteristics, is critical to achieve optimal therapeutic exposure based on bacteria susceptibility and may help to maximize bacteria killing and minimize toxicity [3,121]. This is especially important when dealing with patients at high risk of death and in environments with an escalating resistance to AM.

Promoting high peak concentration for concentration-dependent antibiotics (e.g., aminoglycosides), which is concentrating the daily dose on only one time-point, or, on the contrary, promoting long exposure time, for time-dependent antibiotics (e.g., penicillins), with prolonged or continuous infusions, was proposed to optimize therapeutic success [122]. However, these strategies may be flawed in the presence of PK changes: high concentration peaks may be toxic or, in contrast, inadequately low (especially in patients with a changing Vd), and the time between doses may not be enough to achieve adequate trough concentration. In addition, in the presence of altered clearance, the concentration of time-dependent antibiotics may always be under the adequate target or, alternately, it may accumulate and lead to toxic concentrations [3]. Conventional or nomogram-guided dosing may easily fail to achieve the intended target concentration. This has been demonstrated for vancomycin [123], aminoglycosides [72], daptomycin [124], linezolid [125], and also β-lactams [126,127]. Consequently, an interest in TDM has grown (Table 2).

The potential benefits of TDM are mainly related to the existence of a recognized relationship between serum drug concentration and the intended effect. The same is especially relevant when there is a narrow therapeutic index. Moreover, clinical benefits also rely on the existence of a standardized, easily operated, and unexpensive method to reliably measure drug concentrations, with a high intra and intercenter reproducibility, a short turnaround time (allowing real-time dosing adjustment), and a simplified sampling strategy that facilitates dose adjustment. 

Dosing adjustments based on TDM may be executed in different ways. The most conventional and practical method is based on the evaluation of a single PK sampling at the end of a dosing interval (trough sample, Cmin). This sample is compared against a therapeutic target range, constituting the most popular methodology, albeit also the least accurate for dosing adjustment. Limited sampling strategies incorporate one to three samples, providing more informative concentration–time points for PK description. However, these measurements are only widely available for aminoglycosides and vancomycin. Monte Carlo simulations and PK models can estimate optimal time points. However, dosing nomograms can incorporate measures of organ function and PK/PD data, providing a more accurate method for TDM [15].

The role of routine TDM to optimize antibiotic dosing remains controversial. It was introduced in clinical practice to minimize the occurrence of high trough concentrations and toxicity (especially nephrotoxicity). Along with the increase in antibiotic resistance, the interest in TDM had surpassed the prevention of toxicity goal to include optimizing efficacy, as several animal studies had unveiled a relationship between optimal antibiotic exposure and bacterial killing [128]. This is especially important for aminoglycoside and vancomycin TDM, which are already widely available in most hospitals. The measurement of peak concentration (or AUC calculation) helped to improve the intended target attainment and, consequently, its introduction in clinical practice was straightforward [123,129].

Therapeutic drug monitoring guided therapy facilitates the achievement of pre-specified targets [123,126,127], even in patients undergoing extracorporeal circulation techniques [130,131]. Software that uses a Bayesian approach to dosing that relates clinical and laboratory characteristics of patients, along with careful and timely selected antibiotic concentration measurement, according to drug population PK, is now commercially available, and it may help improve appropriate selection of the dose [132]. An interesting approach to β-lactams PK has also been proposed [133], namely using AG dosing to calculate β-lactams concentration.

Recently published papers strongly advocate using TDM to optimize dosing of AM not only for AG and vancomycin but also for β-lactams, daptomycin, and teicoplanin (along with antifungals and antivirals) [15]. The rapid increase in bacteria resistance to available antibiotics, as well as emerging PK data unveiling that commonly used antibiotic doses may easily lead to low antibiotic concentrations [36,134] as well as the existence of a PK intra-patient variability throughout treatment course [2,135,136], suggest that benefits may extend to these AM. 

Dosing of β-lactams based on antibiotic concentration sampling has been shown to improve target attainment [126,127,136,137]. This is in evident contrast with clinical dosing based only on renal function, which may easily lead to both toxicity or underdosing [138,139], as a hardly predictable ARC [31,140] often leads to underdosing. Nevertheless, the selected therapeutic targets are often dependent on a theoretical MIC value (due to the common absence of microbiological documentation), assuming that the ideal target should be the same for every focus of infection, for different bacteria, and for all patients. All of these assumptions, if proven wrong, may lead to flawed conclusions [141]. Most data supporting these therapeutic antibiotic concentration targets come from retrospective studies [36,75,142,143] and are not properly validated; the relationship between antibiotic guided dosing and effectiveness is dependent on the bacteria susceptibility (MIC), which is difficult and subject to important flaws [144] that preclude precise dose individualization; for the majority of antibiotics, particularly β-lactams, a clear-cut relationship between PK/PD targets and therapeutic outcomes has not been clearly elucidated. Finally, toxicity may not be so clinically relevant [145], and just increasing the dose may easily compensate for the PK changes. Another possible pitfall of relying on antibiotic plasmatic concentration to guide dosing is the possible significant difference between serum and tissue concentrations, which may increase in critically ill septic patients [146,147]. Using microdialysis with real-time feedback information may overcome this limitation [148], but there are no data to support this strategy. If a TDM approach is used, rapid feedback information to the clinician on the bench side is probably useful [149].

In clinical practice, the major benefit of rapid antibiotic bacteria killing seems to be mostly concentrated in the first days of therapy. Using timely administered high antibiotic doses after sepsis diagnosis seems to be of utmost importance. Consequently, avoiding antibiotic delay [150,151], using PK and PD principles to guide antibiotic dosing, such as achieving a high peak of concentration-dependent antibiotics and using extended infusions of time-dependent antibiotics [152] during the first 48 h of therapy, is probably safe and may improve clinical outcomes. After achieving adequate sepsis control, when the patient is improving and the bacteria inoculum is reduced, the prevention of overexposure and potential toxicity [153,154] should also become a priority.

Improving antibiotic concentration throughout time also has the potential for reducing resistance emergence [155,156,157], although higher than commonly used doses may be necessary [158], especially in in vitro models. Higher antibiotic concentrations well above the MIC, throughout the whole course of treatment, not only peak and steady state but also trough concentrations, may be necessary to prevent enrichment of the sample with resistant mutants [159]. Again, preventing a long time of antibiotic exposure and avoiding low antibiotic concentrations may help to decrease the emergence of resistance [160,161]. Reducing the time of antibiotics may also help to decrease adverse events and is probably safe in most settings [162,163].

Prospective, well-designed RCT, supporting the use of TDM and PK/PD targeted therapy, should include real-time MIC data, TDM adequate resources, and focus on patients most likely to benefit from this approach. Studied outcomes should include clinically relevant outcomes along with PK information and emergence of resistance.

## 6. Other Approaches to Optimize Dosing–Nebulization

The increased prevalence of VAP caused by MDR pathogens, the poor lung penetration of commonly prescribed AM, and the absence of new AM in the pipeline led clinicians to search for alternative approaches to optimize drug dosing in the lung, of which the use of inhaled antibiotics is the most used and studied approach [5,164,165,166].

This alternative method is supported by data obtained in animal models as well as in studies with VAP patients. In several animal models, good lung deposition was demonstrated, both in healthy lungs and pneumonia, with amikacin, ceftazidime, and colistin [167,168,169]. The concentration achieved in lung tissue was three to 30 times higher than with systemic IV administration [170]. However, the distribution of the nebulized drug seems to be dependent on the degree of lung disease. In anesthetized piglets on prolonged mechanical ventilation with a model of pneumonia, lung tissue concentrations of various aerosolized antibiotics were markedly higher in pulmonary segments with early stages of lung infection (less consolidated) than in segments with confluent pneumonia and lung abscess [171].

There is also evidence that in VAP patients, nebulization is able to attain higher concentrations in tracheal secretion [172]. In a phase 2 trial using a vibrating mesh nebulizer with a dose of amikacin 400 mg twice daily, only 50% of patients achieved the primary endpoint that was tracheal aspirate amikacin concentration > 6400 μg/mL (which was 25 × 256 μg/mL reference MIC of the most resistant pathogens) [173]. Effective colistin concentrations can also be obtained in tracheal respiratory secretions by inhalation at a dose of 1 MU (80 mg) every 8 h via a vibrating-mesh nebulizer [174].

There are other issues that are also crucial in antibiotic nebulization, such as the solution of the antibiotic (pH, osmolality, preservatives) [175,176,177], the nebulizer device (jet, ultrasonic, static or vibrating mesh), mechanical ventilation settings (volume-controlled ventilation, continuous inspiratory flow, low inspiratory flow to avoid turbulence, low respiratory frequency < 12 bpm, with an I:E of 1:1, with an end-inspiratory pause of 20% of the duty cycle, with patient sedated with Vt 7–9 mL/kg and a minute ventilation <6 L/m, stopping active humidification, since it can increase the particle size), need of deep sedation, and concomitant medication such as bronchodilators [165,172].

The choice of the nebulized antibiotic is also a matter of debate. For example, the choice of aminoglycosides has been challenged because of high levels of resistance in pathogens associated with VAP [178]. In addition, there are data showing that colistin methanosulfate is not a good alternative, since it is a prodrug, the conversion to the active drug in the lung is undetermined, and data have shown high binding to tracheal mucin that could limit distal drug delivery [179]. Some antibiotics, such as ceftazidime and colistin, are also associated with tracheal irritation and cough, causing ventilator assynchrony [169,180].

With all these caveats, it is also important to evaluate the drug deposition in normal lungs and pneumonia with scintigraphic studies performed in patients. In normal lungs, the data point to a reasonably uniform distribution between the left and right lung as well as ventral and dorsal regions [181]. Other studies pointed to a two-compartment model of distribution with an inner and outer region [182]. However, when comparing spontaneous breathing with mechanical ventilation, the latter presents a loss of 20% of drug deposition [182]. Even in normal lungs, there is marked variability between patients and also according to the ventilatory mode (volume-controlled vs. pressure-controlled ventilation) [183]. Using electrical impedance tomography (EIT), it was clear that a patient with pneumonia and consolidation presents an absence of aeration in that area [184]. As a result, the loss of lung aeration as well as the severity and extension of parenchymal infection are all factors that certainly influence the lung deposition of nebulized antibiotics [171].

Nebulized antibiotics have been used with two aims: as an alternative to IV antibiotics and as an adjunctive therapy in addition to IV. The main aim of this strategy is to achieve good antibiotic concentrations at the lung parenchyma, minimizing systemic effects: namely toxicity, antibiotic pressure, and the rate of emergence of MDR pathogens [185]. The use of nebulized antibiotics as an isolated therapy in pneumonia (as an alternative to the IV route) cannot be recommended, since there are no data to support this strategy. Moreover, 10–20% of VAP present secondary bacteremia that would not be adequately treated with this approach [93].

Until 2018, the data on nebulized antibiotics on VAP were scarce and of poor quality, with eight observational studies and eight RCT. However, overall, the use of nebulized antibiotics in VAP patients with MDR pathogens seemed to be associated with better clinical resolution and lower mortality without nephrotoxicity [186,187]. Better clinical resolution was associated with nebulized tobramycin and colistin, not with amikacin, and survival was associated mainly with tobramycin [187].

Even with all of these limitations and knowledge gaps, in a large international survey, only 1/4 of ICUs reported not using nebulized antibiotics [188]. Among those ICU that reported using nebulized antibiotics, 65% used this strategy in ≥ 5 patients in the previous survey month. The major indications were VAP and ventilator-associated tracheobronchitis, the most commonly used device was a jet nebulizer, and the most prescribed antibiotics were colistin methanesulfonate, colistin base, and amikacin [188].

However, in the last few years, three RCT were completed and published using nebulized antibiotics as an add-on to systemic IV therapy assessing different strategies (amikacin with fosfomycin, amikacin alone, and tobramycin) in comparison with placebo in VAP patients that brought light and evidence to the uses as well as the strengths and limitations of nebulized antibiotics [189,190,191].

The IASIS was the first high-quality RCT published to assess the safety and efficacy of the amikacin/fosfomycin inhalation system (AFIS) (300 mg amikacin/120 mg fosfomycin 10 days) for the treatment of GNB VAP. A total of *n* = 143 patients were included (*n* = 71 to AFIS and *n* = 72 to placebo). The course of clinical pulmonary infection score (CPIS), the primary endpoint, from baseline was virtually the same in both treatment groups (*p* = 0.70). In addition, D28 mortality was also not statistically different comparing AFIS vs. placebo (24% vs. 17%, *p* = 0.32, respectively).

For the second RCT, INHALE, a specially designed drug device, was developed (Amikacin Inhale^®^ 400 mg), which is a synchronized inhalation system with the ventilator respiratory cycles. The amikacin solution was a preservative-free solution with adjusted pH to minimize the risk of bronchospasm. The INHALE was a phase 3 superiority RCT conducted in 153 hospital ICUs in 25 countries, in mechanically ventilated patients with GNB VAP [190]. A total of *n* = 725 patients were assigned to standard of care plus Amikacin Inhale (362 patients 400 mf bid 10 d) and aerosolized placebo (363 patients). Both arms presented a similar survival (primary endpoint), 75% and 77%, respectively, as well as a similar rate of adverse events.

Finally, the VAPORISE was a single-center RCT to assess the impact of adjunctive therapy with inhalation tobramycin (twice-daily tobramycin inhalation 300 mg vs. placebo 8 days) in VAP patients [191]. The trial was terminated prematurely due to lack of inclusion with only *n* = 26 patients (tobramycin *n* = 13, control *n* = 13). The 30-day mortality was the same in both arms (*n* = 4, 31% in both); there were also no significant differences concerning treatment failure (primary endpoint and ventilator-free days (VFD).

All these RCTs had some potential problems that could have impacted outcomes. It is important to stress that nebulized antibiotic as an adjunctive therapy to IV route is only used while the patient is under mechanical ventilation, and this could be associated with a longer duration of mechanical ventilation and delayed extubation to complete nebulization therapy [177,180]. That was the case in the IASIS trial with less VFD in the intervention arm, but not in the INHALE trial with a similar duration of mechanical ventilation in both groups. In addition, the risk of expiratory filter occlusion is well known in patients receiving nebulized therapy with vibrating mesh devices. This is an area of concern that has not been systematically evaluated, but it is known to interfere with ventilation with one described case of cardiac arrest [180].

There are also some potential benefits from this approach, namely the decrease in the emergence of MDR pathogens. Since the epithelial lining fluid concentrations attained with nebulization are frequently well above MIC, such high levels in the lung might contribute to decreasing the risk of emergence of drug resistance [192]. Although controversial, this has been suggested in two studies in patients with ventilator-associated tracheobronchitis [185,193] but without robust data from the recent RCTs.

As a result, currently, there is no evidence to support initial nebulized antibiotics in combination with intravenous therapy in intubated, mechanically ventilated patients with VAP [94,177,194]. However, in three SRMA, it seems that in GNB VAP caused by pathogens only susceptible to aminoglycosides and polymyxins, adjunctive antibiotic therapy with colistin led to better outcomes [195,196,197]. Nevertheless, the results from the IASIS and INHALE trials challenged the findings of these SRMA, since the survival and cure rates of patients with MDR and XDR pathogens were similar to those without MDR [189,190].

Taking into consideration current evidence, the recommendations of ATS/IDSA guidelines seem reasonable [93], since the use of nebulized antibiotics is restricted only in VAP caused by PDR and XDR pathogens in whom treatment options are limited to intravenous antibiotics with poor lung penetration (for example, colistin and aminoglycosides) or with systemic toxicities preventing increases of intravenous dosing. Finally, future studies are needed to evaluate the impact of this rescue therapy on outcomes as well as to define the best delivery device [166].

## 7. Impact of Dosing Strategies on Outcomes

**Figure 2:**
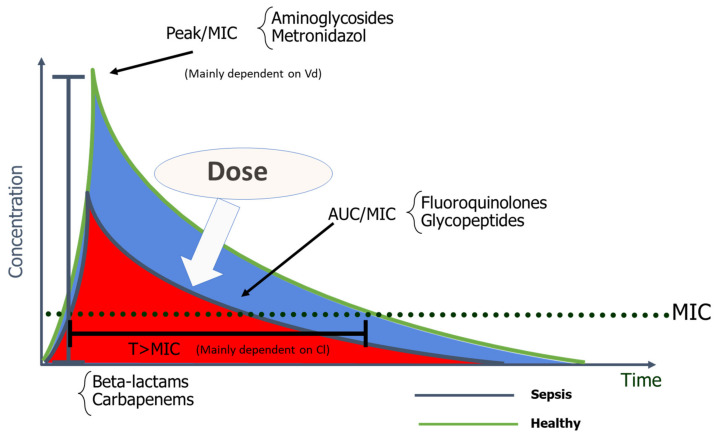
Pharmacokinetics and pharmacodynamics: After an antibiotic infusion, a ratio between the peak concentration and the minimal inhibitory concentration, the area under the concentration–time curve and the minimal inhibitory concentration, and the time the antibiotic concentration is above the minimal inhibitory concentration can be defined. These three parameters all decreased in septic patients, with an increased volume of distribution and clearance. The only secure way to achieve effective antibiotic concentration is to adjust the dose. MIC—minimal inhibitory concentration; T > MIC—time that the antibiotic concentration is above the minimal inhibitory concentration; Vd—Vole of distribution; Cl—Clearance.

Bacteria killing by antibiotics is closely linked to exposure. According to the PK/PD relationship, antibiotics’ killing is mostly related to the time above bacteria MIC (T > MIC) or peak concentration/bacteria MIC (Peak/MIC) (Figure 2). In a series of landmark studies, ideal exposure was calculated as T > MIC of roughly 40–60% (for β-lactams), ratio of AUC/MIC of 30–40 (Gram positive) or 120 (GNB) for fluroquinolones, and Peak/MIC 8–10 for aminoglycosides [10,35,128]. However, the majority of these studies rely mostly on retrospective data, and in vivo evidence is still poor and sometimes conflicting, especially when referring to β-lactams [198,199].

It should also be noted that in clinical practice, patients with renal failure, a well-known risk factor for worse outcome and mortality, often easily achieve higher and prolonged antibiotic exposure [137,200,201]. Consequently, there may be a significant interaction between this high antibiotic exposure and the high-risk clinical status, which may limit the evaluation of the potential benefit of adequate antibiotic exposure. Moreover, bacteria killing is a biologic (and not mechanical) process and significant inter-patient and intra-patient in vivo variability may be noted [202,203].

Optimization of dosing strategies for each particular antibiotic in any single patient may help to improve clinical and microbiological outcome. Increasing the time of exposure of β-lactams as well as achieving high exposures of fluoroquinolones and peak concentrations of AG are associated with optimal bacterial killing in vitro [16,35].

In critically ill patients, higher targets have been proposed, especially for β-lactams, including a time of 100% over a concentration as high as 4*MIC [143,201]. Consequently, an extended or even continuous infusion of these antibiotics has been proposed as able to achieve these higher targets and improve clinical outcomes.

In a before–after study, Lodise et al. [204] evaluated a program to improve piperacillin/tazobactam dosage, with the use of extended infusions to treat infections caused by Pseudomonas aeruginosa. A decrease in mortality was noted, mostly in patients with the more severe infections and higher risk of death. However, the same benefit was not found by Gonçalves-Pereira et al. [205]. In this propensity score-matched population, mortality was the same whether continuous or intermittent dosage of piperacillin/tazobactam was used. It is important to note that in this last study, higher doses of piperacillin/tazobactam were used: 18 g/day in both arms [205].

An eventual benefit of using continuous (or extended) infusions of β-lactams was the subject of several meta-analyses. Although some small differences were noted according to the studies selected, two of the more comprehensive studies, including a Cochrane meta-analysis, did not find any difference in the outcomes, including all-cause mortality, infections recurrence, super-infection, or adverse events [30,206]. This highlights the complexity of infection treatment and the difficulties of precision medicine, that is to say, choosing the ideal dose for each patient at a particular time point [3].

A large multicenter point-prevalence observational study [207] was performed to evaluate the appropriateness of antibiotic dosing in clinical practice as well as the clinical outcomes. A practical simplified PK evaluation was selected with only a two-point sampling during one dose interval. In the sub-group of patients treated for infection (*n* = 248), 16% did not achieve the intended target of 50% free T > MIC, and these patients were less likely to have a positive clinical outcome (OR 0.68). On the contrary, an improved clinical outcome was noted in patients who achieved the same target concentration or even 100% free T > MIC ratios (OR 1.02 and 1.56, respectively). Of note, evaluation of the targets 50% and 100% free T > 4*MIC did not show any additional benefit [207].

Three prospective RCT assessed β-lactams dosing, comparing continuous infusion with intermittent conventional dosing [47,208,209]. In all three RCT, patients receiving RRT were excluded. The first RCT [208] included 140 patients and found a better clinical cure rate in patients receiving continuous infusion β-lactams (56 versus 34 %, *p* = 0.011), along with higher rates of the intended target (100% free T > MIC) attainment on day 1 (97 versus 70 %, *p* < 0.001). Nevertheless, there were no differences in 14 or 30-day mortality. The second RCT [209] enrolled 60 patients. At day 3, there was a PK and clinical benefit for the continuous infusion group, with plasmatic antibiotic concentrations above the MIC in 82% of patients vs. 29% in the intermittent group (*p* = 0.001). In addition, a higher rate of clinical cure was noted (70% vs. 43%; *p* = 0.037), but again, no survival benefit was found at hospital discharge (90% in the continuous group vs. 80% in the intermittent group, *p* = 0.47). The third, a larger RCT [47] including 432 critically ill infected patients (APACHE II of 20) found no difference in the clinically relevant outcomes, namely ICU-free days, 90-day survival (74.3% vs. 72.5%; *p* = 0.61), clinical cure (52.4% vs. 49.5%; *p* = 0.56), organ failure-free days, or duration of bacteremia.

A meta-analysis of the individual data [210] of these three RCT pointed to a small, although significant, clinical benefit of continuous infusion, hospital mortality (19.6% vs. 26.3%, RR 0.74 (95% CI 0.56–1.00), *p* = 0.045) and clinical cure (55.4% vs. 46.3%; RR 1.20 (95% CI 1.03–1.40), *p* = 0.021). Consequently, a larger multicenter RCT with a similar protocol, addressing 90-day all-cause mortality, intending to recruit 7000 patients, is currently underway [211]. Assuming that PK is an important determinant of infection resolution and clinical outcome, another possible strategy is the use of TDM to guide dosing and ensure adequate antibiotic exposure.

An observational study addressed the clinical use of seven different β-lactam antibiotics TDM guided therapies (with HPLC) in 369 infection episodes [51]. A need to change the selected initial dose was noted in roughly half of the patients (33.1% need a dose increase to achieve a target of 100% free T > MIC and 17.3% need a dose reduction being above 100% free T > 10%MIC). No significant clinical impact of early achievement with this intended PK/PD target was found (OR 0.88 (95% CI 0.40–1.91); *p* = 0.74). The outcome was mostly related to classical risk factors (an abdominal source of infection, higher prognostic scores). It is important to note that except for ampicillin, more than 90% of patients achieve the more conservative target of 50% free T > MIC [75]. In this study, ARC (defined as ClCr >130 mL/min) was the main risk factor for underdosing (OR 2.47), but the same is also linked to better baseline organ functional status and a possible better prognosis [212].

Similarly, Richter et al. addressed a TDM program of continuous infusion of piperacillin/tazobactam (target free 100% T > 2–4*MIC) in 484 patients [137]. After the initial dose, the PK target was attained in 34.3%, and low concentrations were noted in 10.1%. After dose adjustment, target attainment was reached in 62.4% of patients. In this study, a mortality benefit was associated with antibiotic concentration after the first dose. Both low concentration and toxic levels were associated with significantly higher mortality (20.8% and 29.4%, respectively) compared to 13.9% in those on target (*p* < 0.005) [137].

A stronger relationship between antibiotic exposure and outcomes has been shown for AG and vancomycin.

For AG, optimal bacteria killing is related to the ratio between the peak concentration and the MIC [213]. In contrast, nephrotoxicity is related to trough concentration, and renal antibiotic accumulation is dependent on a saturable mechanism with a Michaelis Menten kinetic. Consequently, using higher doses but with large intervals (a “once-daily dose”) is recommended to improve outcomes and prevent toxicity [129]. A peak concentration as high as 20 mg/L for gentamicin and tobramycin (50 mg/L for amikacin) has been proposed for treating hospital-acquired infections, and an initial dose of at least 7 mg/kg of gentamicin was recommended to achieve these high concentrations [142]. However, even these elevated antibiotic doses may not suffice to account for the augmented Vd, and higher doses may be necessary [72,73,214]. For these antibiotics, once daily TDM-guided dosing has been proposed to improve outcomes [215]. However, in patients with renal failure, larger intervals may be necessary to prevent renal accumulation and toxicity [216] and also to decrease the risk of adaptative resistance, which is mediated by the overexpression of efflux pumps after overexposure to aminoglycosides [213].

In clinical practice, two concentration measurements, a peak sample and a second one collected during the elimination phase [217], provide enough information to calculate PK-guided dosing, either with the use of a Bayesian dosing software or with the Sawchuk and Zaske formulas [218]. It should be noted that even these high peak concentrations may not prevent the emergence of resistance [160] and the concentration–time AUC may also be important.

Concerning vancomycin, there are also some data, mostly retrospective, correlating vancomycin exposure with clinical outcome. Moise-Broder et al. [148] identified a relationship between an AUC (24 h)/MIC > 400 and therapeutic success in hospitalized patients with MRSA pneumonia.

However, AUC is not easily measured in clinical practice, and trough concentrations are commonly used as a surrogate [219]. Nevertheless, the easiest and safest way to achieve this PK target is the use of continuous infusions [220], with most data suggesting equivalence in outcomes with intermittent dosing [84]. This dosing strategy may also help to prevent nephrotoxicity [221] and decrease the emergence of resistant bacteria [75].

When using vancomycin by continuous infusion, according to published available PK data, it is recommended to use a loading dose of 15 to 20 mg/kg, followed by daily maintenance dose of 30 to 40 mg/kg (up to 60 mg/kg), guided by steady-state TDM, to achieve a target concentration of 20 to 25 mg/L, especially in critically ill patients. This should provide an AUC (24)/MIC of 480–600, assuming a MIC of 1 mg/L. Of note, this strategy has never been prospectively validated [75]. 

The limited available data concerning the relationship between PK/PD targets and outcomes should not be viewed as proof of lack of benefit. Absence of evidence of benefit does not equal evidence of absence of benefit. While the attainment of any antibiotic concentration target does not guarantee per se efficacy or an improved outcome for any single patient, using individually guided dosing optimizes the probability of achieving a numerically higher favorable response in a whole population [202]. Variability in targets (such as the organism’s MIC) can be considered in models with subsequent adaptation as new information arrives. That is, complexity alone should not relegate the decision-making process to clinician guessing. The exposure–response relationship is necessarily complex, and it is modified by patient, bacteria, and the focus of infection-specific factors. Information regarding precision dosing should inform clinical decision making rather than protocolize it in an absolute mode [202].

## 8. Future Directions and Conclusions

Several factors contribute to the success of infection management in critically ill patients. In addition to the adequacy of empiric AM therapy, optimizing dosing is also crucial, namely the prescribed dose, the route, and the mode of administration. This is particularly challenging in critically ill patients due to PK changes related to the unpredictable Vd as well as degree of organ failure, both renal and liver, but also ARC. As a result, to attain target concentrations, the current recommendations point to higher doses of AM in the first days with TDM whenever possible.

Future research should address some unmet needs, namely TDM of different AM, as well as defining target concentrations, assessing the correlation between serum and tissue concentrations of AM, optimizing AM dosing during the course of the infection and according to disease severity, among others, that could improve several clinical outcomes in an era of increasing MDR pathogens.

Meanwhile, we must use the available tools to optimize individual AM dosing to maximize the exposure and effectiveness of these drugs in critically ill patients.

## Figures and Tables

**Table 1:** Volume of distribution of ICU antibiotics.

Antibiotics that Stay in Extracellular Fluid (Vd < 0.3 L/kg)	Drugs that Distribute into Total Body Water (Vd 0.7–1 L/kg)	Drug with High Distribution to Tissues (Vd > 1 L/kg)
- Aminoglycosides - Beta-lactams - Penicillins - Cephalosporins - Carbapenems - Daptomycin	- Clindamycin - Linezolid - Metronidazole - Vancomycin	- Colistin - Fluoroquinolones - Macrolides - Azithromycin - Clarithromycin - Tigecyline

**Table 2:** Pharmacokinetic and pharmacodynamic characteristics of common antibiotics in intensive care medicine [15].

Antimicrobial Class	Monitoring/ Sampling	PK/PD Target	Toxicity Threshold
Therapeutic Drug Monitoring Recommended			
**Beta-lactams** - Penicillins - Cephalosporins - Carbapenems	Cmin/One sample ^1^ Css (continuous infusion)/One sample ^2^	100% fT> MIC Css > MIC 50–100% fT > MIC 45–100% fT > MIC 50–100% fT > MIC	Nephrotoxicity/Neurotoxicity Cmin > 361 mg/L (Piperacillin nephro-/neurotoxicity) Cmin > 20 mg/L (Cefepime neurotoxicity) Cmin > 44.5 mg/L (Meropenem nephro-/neurotoxicity)
**Aminoglycosides** - Gentamicin - Amikacin	AUC-based/Two samples ^3^ Cmax/MIC/One sample ^4^ Cmin/One sample ^1^	AUC 80–120 mg h/L Cmax/MIC ≥ 8–10 Cmin <0.5 mg/L Cmin <2.5 mg/L	Nephrotoxicity/Ototoxicity Cmin> 1 mg/L Cmin> 5 mg/L
**Glycopeptides** - Vancomycin	AUC/MIC/Two samples ^5^ Cmin/One sample ^1^ Css/One sample ^2^	AUC (0–24)/MIC ≥ 400 Cmin ≥ 15–20 mg/L ^6^ Css 20–25 mg/L	Nephrotoxicity Cmin > 20 mg/L
Therapeutic Drug Monitoring Neither Recommended nor Discouraged			
**Colistin**	Cmin/One sample ^1^ AUC (0–24)/MIC	Cmin 2 mg/L Not defined	Nephrotoxicity Cmin > 2.4 mg/L
**Fluoroquinolones**	AUC/MIC/Two samples ^7^ Cmax/MIC/One sample ^4^	fAUC0–24/MIC ≥ 80 Cmax/MIC ≥ 8–12	Not defined

^1^ 30 min or just before next dosing. ^2^ One sample at any time point during the infusion. ^3^ One 30 min after the end of infusion and another 6–22 h after infusion. ^4^ 30 min after the end of infusion. ^5^ 1 h after the end of infusion and another within 1–2 h of the next infusion. ^6^ For severe infections. ^7^ 2 h after dosing and the other 6 h after dosing; Cmin trough drug concentration; MIC minimum inhibitory concentration; fT > MIC percentage of time over 24 h that the free antimicrobial concentration exceeds the MIC; Css average steady-state drug concentration; AUC area under the concentration–time curve; Cmax/MIC ratio of maximum drug concentration to minimum inhibitory concentration; AUC/MIC ratio of the area under the concentration–time curve during a 24 h period to minimum inhibitory concentration.

## Data Availability

No new data were created or analyzed in this study. Data sharing is not applicable to this article.
